# Spatial Heterogeneity of Autoinducer Regulation Systems

**DOI:** 10.3390/s120404156

**Published:** 2012-03-28

**Authors:** Burkhard A. Hense, Johannes Müller, Christina Kuttler, Anton Hartmann

**Affiliations:** 1 Institute of Biomathematics and Biometry, Helmholtz Zentrum München, Ingolstädter Landstraße 1, 85764 Neuherberg, Germany; 2 Department of Mathematics, Technische Universität München, Boltzmannstraße 3, 85748 Garching, Germany; E-Mails: johannes.mueller@mytum.de (J.M.); kuttler@ma.tum.de (C.K.); 3 Research Unit Microbe-Plant Interactions, Helmholtz Zentrum München, Ingolstädter Landstraße 1, 85764 Neuherberg, Germany; E-Mail: anton.hartmann@helmholtz-muenchen.de

**Keywords:** autoinducer regulation network, spatial heterogeneity, division of work, hybrid push/pull control, quorum sensing

## Abstract

Autoinducer signals enable coordinated behaviour of bacterial populations, a phenomenon originally described as quorum sensing. Autoinducer systems are often controlled by environmental substances as nutrients or secondary metabolites (signals) from neighbouring organisms. In cell aggregates and biofilms gradients of signals and environmental substances emerge. Mathematical modelling is used to analyse the functioning of the system. We find that the autoinducer regulation network generates spatially heterogeneous behaviour, up to a kind of multicellularity-like division of work, especially under nutrient-controlled conditions. A hybrid push/pull concept is proposed to explain the ecological function. The analysis allows to explain hitherto seemingly contradicting experimental findings.

## 1. Introduction

Extracellular signalling via small diffusible compounds (autoinducers) is used by many bacterial species, including pathogens, mutualistic as gut commensals, and plant growth promoting bacteria. In brief, bacteria release autoinducers and simultaneously regulate target gene expression dependent on the environmental autoinducer concentration [1]. Most autoinducer systems include a positive feedback loop, often by autoinducers' control of their own production. In combination with nonlinearity (e.g., using oligomers of autoinducer-receptor complexes as active units), this often results in switch like regulative behaviour. Functionality of various autoinducer systems has been investigated in a number of mathematical modelling approaches [2]. Autoinducer regulation was originally assumed to be a strategy enabling synchronous and uniform life style switches of the whole bacterial population in dependency on the cell density. This strategy has been termed “quorum sensing” (QS) [3].

Within cell aggregates like biofilms or colonies, spatial gradients of autoinducers may emerge and result in a spatial organisation of autoinducer induction. Spatially heterogeneous induction of autoinducer regulated genes has indeed been found in some biofilm experiments. Model results suggest that the highest autoinducer concentration is always present in the colony centre or near the attachment surface of biofilms. However, different experimental studies reported strongest upregulation of autoinducer controlled genes both, near the bottom or at the top of biofilms, partly even for the same species [4–6]. These contradictions have not been explained, yet.

Based on more indirect arguments, the few biofilm models, which include a nutrient/autoinducer connection, assume that a decreasing nutrient supply downregulates autoinducer activity [7–9]. Seemingly, this assumption is appropriate for some species. However, autoinducer induction often regulates responses to stress as starvation, and the scare existent quantitative analyses suggest non-linear or even non-monotonous relations ([10], Mellbye and Schuster, pers. comm.). At least in a certain range, nutrient deficiency promotes autoinducer activity. Necessarily, at very severe starvation autoinducer production will finally abolish. Potential nutrient gradients in cell aggregates/biofilms can affect further the spatio-temporal heterogeneity of autoinducer regulation.

**Figure 1.**
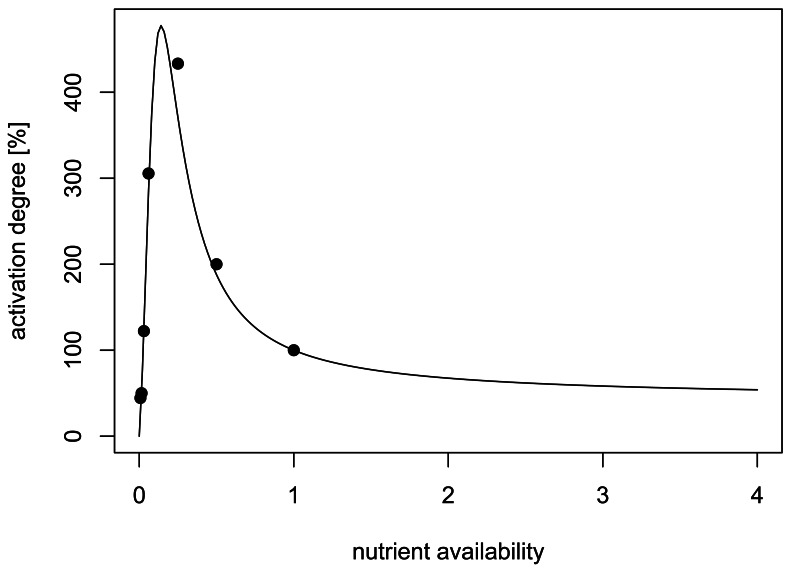
Fit of modulation of the AHL system by nutrient availability. Dots are data from [10], the solid line indicates the fit. Activation degree denotes the relative autoinducer production: production rate with standard medium was set to 100%. Nutrient availability: dilution of standard medium. For more details of the fitting model see Section 4.1.3.

We developed a 3D model of autoinducer regulation in attached microcolonies based on a typical *lux-type* autoinducer system. The relation between nutrient and autoinducer production is integrated as experimentally reported in [10], which presents to our knowledge the best quantitatively analysed system (Figure 1). The main objective of our study was to develop a model for a mechanistic analysis of the developing spatio-temporal heterogeneity of autoinducer systems in growing microcolonies, with a special focus on the influence of nutrients as an example for interacting environmental factors in order to explain the seemingly contradicting experimental findings. We will further discuss the potential ecological implications: Which information is sensed via autoinducers and what is the benefit?

Although mainly based on data of *lux* autoinducer system in *Vibrio fischeri*, this study is not meant to provide a quantitative analysis of autoinducer regulated activities in *V. fischeri*, but to contribute generally to the development of the theory of autoinducer regulation under complex environmental conditions.

## 2. Results and Discussion

We consider a generic model focusing on the interaction between the autoinducer system and nutrient availability, under consideration of emerging gradients of both in a bacterial colony. Our main hypothesis is that spatial heterogeneity is mainly caused by an interplay of these two systems.

### 2.1. Model

The cells we model possess an autoinducer system of lux-type with an AHL (acylhomoserine lactone) acting as an autoinducer. It binds to a receptor molecule (LuxR), the AHL-receptor dimerises. The dimers bind to the *lux* operon, where the autoinducer synthase (LuxI) and luminescence genes are up-regulated, but also to other target genes of the regulon [3]. We assume that the bulk fluid is large, so that no accumulation or depletion of released or consumed substances occurs. Loss of AHL is mainly due to diffusion, whereas degradation by lactolysis can be neglected under slightly acid conditions [11]. We focus on equilibrium situations, which seems reasonable as diffusion and change of induction state occur at faster time scales than, e.g., microcolony growth.

We give a brief description of the mathematical model. We start with the spatial geometry. Subsequently we address nutrient *N*(*x, t*) availability and AHL *A*(*x, t*) regulation (see Table 1).

#### 2.1.1. Geometry

Let us consider a colony, whose fraction of volume occupied by cells is given by *ρ*(*x*). Note that we do not measure the number of cells per volume (cells per volume), but the fraction of the volume occupied by cells. Of course, both data are equivalent.

The colony is centred around the origin of the three-dimensional space. We do not consider population dynamics: As population dynamics is slower than the processes we shall consider here, we are allowed to assume the population size to be fixed; the results of a growing colony resemble the results we obtain here. Furthermore, a rotationally symmetric setting is assumed, *i.e.*, *ρ*(*x*) = *ρ*(|*x*|). The colony has radius *R, ρ*(|*x*|) = 0 for |x| > *R*.

#### 2.1.2. Nutrient

*N*(*x, t*) is a generic replacement of all nutrients we ever need. We do not relate *N*(*x, t*) to the concentration of a specific nutrient like glucose. This nutrient is available in 100% (corresponding to *N* ≡ 1) if we go arbitrary far away from the colony. Within the colony it is consumed in a Michaelis–Menten way, generating nutrients gradients in dependency of diffusion properties.

$$\begin{array}{l} \;N_{t}=D_{N}\Delta N-\rho \left(x\right)\frac{K_{\text{cat},N}N}{N+K_{m,N}}\\ \begin{array}{cc} N\left(t,x\right)\to 1 & \text{for}\left|x\right|\to \infty \end{array} \end{array}$$

#### 2.1.3. AHL

Diffusion of AHL is formulated in a standard way. Only the production term of AHL is interesting. Following the usual modelling approach [12], we use a constant production rate to indicate the basic expression, and a Hill function that corresponds to the increased production rate in the induced state. The Hill coefficient specifies the cooperative effects of the regulatory pathway controlling the autoinducer production. The complete production rate is multiplied by the cell concentration. Additionally, it is also multiplied with a factor *f*(*N*), which reflects the modulation of the signalling system by nutrient availability.

$$\begin{array}{l} \;A_{t}=D_{A}\Delta A+\rho \left(x\right)\;f\left(N\right)\left[\alpha +\beta \frac{A_{n}}{A^{n}+A_{\tau }^{n}}\right]\\ \begin{array}{cc} A\left(t,x\right)\to 0 & \text{for}\left|x\right|\to \infty \end{array} \end{array}$$

The rational of parameter selection and numerical methods for simulation can be found in Section 4.

### 2.2. Effect of Nutrient Availability on AHL Regulation

**Figure 2.**
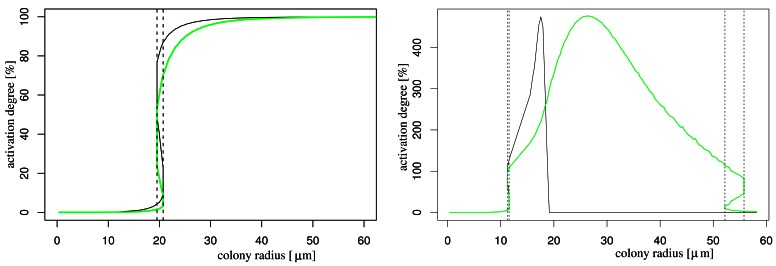
Bifurcation diagram without (left panel) and with (right panel) nutrient influence. Black curve: activation degree in the centre of the colony. Green curve: activation degree at the boundary. Vertical lines indicate intervals where bistability happens.

In order to understand the effect of nutrient availability on AHL production, we simulate the system with and without influence of nutrient on the AHL production (for the latter we take the function *f*(*N*) to one). In both cases, we obtain a well-known bifurcation diagram for systems with hysteresis: if the population size is above a critical number (this is, if the colony radius is above a critical magnitude) the colony becomes activated (Figure 2). This critical radius is decreased by the influence of nutrient depletion as it is to be expected. The model that does not incorporate effects of nutrient depletion shows that the activation degree stabilises for larger colony radii, while the model that takes into account nutrient depletion, the activation degree breaks down (due to starvation effects), starting with the central cells (black curve). Eventually also the boundary cells (and therewith the complete colony) starve so much that it is completely deactivated (green curve). This latter behaviour can change if nutrients are continuously delivered, e.g., by flow in the bulk fluid (not shown).

**Figure 3.**
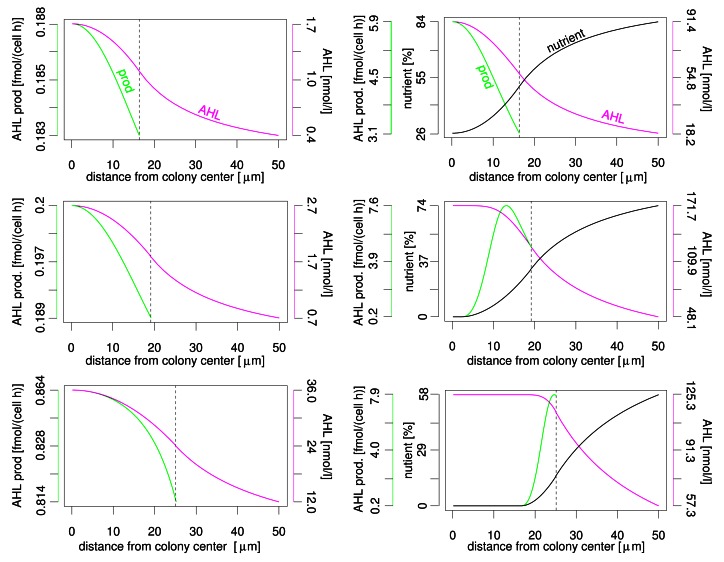
Profiles of autoinducer production (green curve), autoinducer concentration (magenta curve) and nutrient concentration (black curve) in colonies of different sizes (vertical line); left: without nutrient influence, right: with nutrient influence.

Even more interesting is the profile of nutrient, AHL concentration and AHL production rate over the distance to the colony centre (see Figure 3). In both nutrient scenarios the AHL concentration reaches its maximum at the colony centre and declines towards the colony border. The variation of AHL concentration between different locations within the colony is limited (please note that the *y*-axis does not start at zero). *In vivo*, limited gradients may be covered by, e.g., additional cellular regulation pathways, spatially heterogeneous biofilm structures and noise. Variation of AHL production is less than 10% without nutrient influence. Again, the highest production rate is found in the colony centre. But the AHL production rate varies significantly if the nutrient dependency of this rate is taken into account. This applies both for the difference between maximum and minimum production rate and the location of the maximum. The reason is the nutrient depletion that naturally starts at the centre of the colony. Depending on colony size, the maximum activation may be at the colony centre, somewhere between colony centre and colony boundary, or at the colony boundary. If, e.g., AHL production and luminescence (or other target activities) are connected, i.e. both under similar control of autoinducer and nutrients, a stripe of active cells moves from the centre to the colony boundary in case of a growing colony. We expect the same behaviour for different geometrical setups as flat layers of bacteria with varying thickness in a plane biofilm. This observation may explain the observed conflicting experimental results about spatial autoinducer regulation. First/strongest induction in autoinducer systems was reported near colony centre resp. at the biofilm bottom but also at/near upper surface [13–15]. Simple models of autoinducer regulation without influence of additional factors as nutrients usually predict highest activity only in the centre. Interestingly, for autoinducer regulation in *Pseudomonas aeruginosa* [4–6], for which highest autoinducer regulation activity was reported to occur at the top or at the bottom of biofilms, indeed an nutrient influence was experimentally shown (Mellbye and Schuster, personal communication).

The intention of this paper was to promote the general understanding of the analysed regulative subsystem, not a complete, quantitative analysis of a specific strain of *V. fischeri* or another species. Autoinducer systems of other species may have other parameter values, but the general trends will hold. Higher AHL production rates per cell, higher cell densities within the colony or lower threshold concentration for the induction will decline the colony size needed to induce the system. A lower Hill factor, as in a system without multimerisation of LuxI/AHL complexes, decreases or destroys the bistable range. The relation between nutrients (or other environmental factors) and the autoinducer system is critical for the potential of heterogeneity development. Unfortunately, direct quantitative data are largely missing. More complex biofilm morphologies, e.g., channels and density differences between the border and the centre of the colony, and intercellular variability will complicate the activity pattern, but are not assumed to change the conclusions of this paper. Cheaters, which do not produce AHL but respond on it, reduce the average AHL production rate per cell and thus the local AHL concentration, respectively increase the colony size required to reach a certain AHL concentration.

### 2.3. Ecological Interpretation

The main idea about the purpose of the autoinducer system is efficiency (cost effectiveness): The cell regulates relatively expensive actions via the autoinducer system. Compared to the regulated activities, the autoinducer molecules are quite cheap to produce, as they are small and usually active in low concentrations. They are used as a proxy in order to decide if it is worth to trigger these expensive activities. It is straight to understand that the concentration of autoinducer molecules is able to predict the concentration of for example exoenzymes. Thus, it is sensible to use autoinducers to regulate the exoenzyme production. The initial interpretation has been quorum sensing (how large is the population?), later accompanied by diffusion sensing (how large is the diffusible space or, more generally, what are the mass transfer properties?) [3,16]. These two concepts have been unified by efficiency sensing (is it efficient to start any action?) [17].

How is it possible to integrate the observation that nutrient availability (and perhaps also other environmental or intracellular conditions) affects autoinducer production into these interpretations? The idea is that the cells do not perform pure quorum/diffusion/efficiency sensing (an impartial exploration of the space), but, up to a certain degree, they also integrate information about their individual demands into the communication signal and respond to it.

Formally, this is reflected in the model by the dependency of the AHL production on the nutrient function f(N) (see Section 4.1.3). A complete analysis about the effect of nutrients with respect to the efficiency of the regulated activities goes beyond the scope of this paper. However, we can draw some conclusions. In case of nutrient depletion, the increase of autoinducer production can be interpreted as kind of an emergency call. Starving cells may have an increased demand for a coordinated behaviour improving the supply of a limiting nutrient. The demand is communicated by increased autoinducer release. This reduces the number of required cells sufficient to reach the induction threshold. On the other hand, in a spatially structured population the outer cells of the colony, which are yet under less starvation stress, will contribute to the cooperation and thus improve also the conditions for the more stressed inner cells. The system allows for an efficiency optimised regulation on colony level by integration of the specific demands of each cell at its specific side into a spatially structured communication. If the population is very small, an emergency call will not have any effect—though there is an increased autoinducer production, the AHL concentration does not become supercritical. However, with growing colony size the call will be taken up by the colony. Also outer, well-fed cells of this colony start to produce exoenzymes such that the inner, starving cells are supplied with nutrient. Once the starvation becomes too severe, eventually the demand stops.

From the efficiency point, increase of autoinducer signal release under starvation, which implies increased costs, is reasonable in spatially structured populations where cells develop different demands. In mixed plankton, every cell experiences the same nutrient concentration, thus a direct co-control of target genes by autoinducers and nutrients seems more cost effective. Spatial organisation of activity via autoinducers adds a new layer of cooperativity to this communication systems. This observation raises interesting questions about its functionality under the aspect of kin respectively group selection.

**Figure 4.**
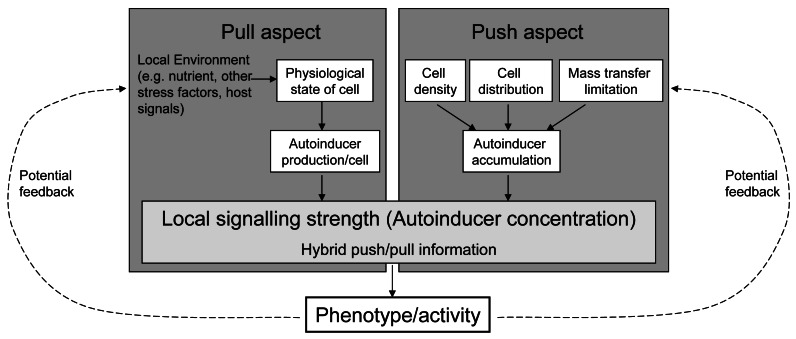
Scheme of the push/pull regulation. Autoinducer regulation systems integrate information affecting the cells demand (pull aspect) with those about the potential cooperative strength of the regulated activity (push aspect; dependent on cell density, cell distribution and mass transfer limitations) into local autoinducer concentration. The regulated phenotype can influence both the pull aspect (for example if the availability of nutrients is increased by exoenzymes) and the push aspect (for example if a changed migratory behaviour influences the cell density or distribution).

Referring to the terminology in industrial production processes and following its recent transfer to biological processes, we propose the term “hybrid push/pull control” for this kind of regulation design ([18,19]). Roughly, “pull” in industrial processes (the actual demand of the buyers for a product) reflects the demand of a cell for the target behaviour, transported by a changing autoinducer production. “Push” in industrial processes (the (potential) strength of a company to produce this product) reflects the possible reached strength of the target behaviour. As the pull and push factors both influence the cost-effectiveness of the regulated activity, we hypothesise that the core purpose of this regulation system is to promote a gene control based on pre-assessment of the efficiency of the target gene resp. behaviour (Figure 4).

Beside nutrients, other intra- and extracellular factors affect the efficiency. Examples are other environmental factors (like pH, temperature), presence of competing or beneficial microbes, indicators for health state of hosts (relevant for potential pathogens), and the developmental state of a cell (like sporulation, mobility). From the efficiency point of view all these factors affect the actual need of the cells for the regulated target behaviour, their potential to contribute to it, or the opportunity that the regulated behaviour is effective (because, e.g., the host is weak). In fact, some of these aspects are known to be connected with autoinducer regulation pathways [20–22]. One idea in that context is that sensing the combined pull/push information carried by the autoinducers allows for a contextual interpretation of the state of the neighbouring cells relative to its own and of the push factors for each cell [23]. This results in an adaptive behaviour of cells within the growing colony, highly dynamic in space and time. Cooperativity and division of work can emerge. Their spatio-temporal flexibility goes beyond those of real multicellular organisms, e.g. because the cell differentiation with respect to division of work can be highly reversible. This remarkable phenotypic plasticity probably enables an adaptive life style optimisation of the entire colony under the current conditions with respect to the fitness.

## 3. Conclusions

A mathematical model was developed interlinking spatially an autoinducer system with an the environmental factor (nutrients). The modelling results indicate that autoinducers can promote a highly adaptable, spatial phenotypic heterogeneity in spatially structured populations, e.g., within colonies and biofilms, instead of inducing homogeneous behaviour of the population. The differentiation into subpopulations, which can be interpreted as division of work, is amplified by the interplay with other regulative factors as nutrient supply. In contrast to heterogeneity caused by stochastic noise [24,25], this can only emerge in spatially structured populations, not in plankton.

Beside push information about the potential strength of a regulated activity, integration of nutrient starvation or other environmental and physiological factors introduces a pull (demand) dimension into the information carried by autoinducers. We hypothesise that the aim of this autoinducer regulation architecture is to ensure the most efficient responses of the whole cell aggregate to changing environmental conditions, aiming to optimise the fitness of the whole colony. Environmental autoinducer concentrations thus carry a highly integrative information, optimised to allow for meaningful decisions of cells in their specific environment.

We think it will be worthwhile to re-consider some biological questions from the perspective of the push/pull concept: The existence of multiple autoinducer systems in some species is more comprehensible by assuming that the efficiencies of different target behaviours depend on different independent pull aspects, varying over time and space. Multiple autoinducer systems allow for independent transportation of such different pull information. Note that the push information might not differ in this case. Non-inducibility via externally added autoinducer in certain growth phases could be connected with downregulation of autoinducer systems (e.g., autoinducer receptors) via pull control [26,27]. The role of the universal autoinducer AI-2 has been questioned—among others—due to its origin as an unavoidable waste product reflecting the activity of the methyl cycle pathway, whereas “true” autoinducers should simply reflect the presence of a cell [28]. However, with respect to the discussion given in this paper, the differences diminish. Temporal pattern of autoinducer controlled gene expression in batch cultures [26], which is believed to be connected to changes in the concentration of, e.g., nutrient and waste product, is *in situ* probably at least partly reflected by spatial patterns. The relevance of spatial structuring affects the development of treatment strategies for pathogens or beneficial bacteria on autoinducer level: For instance, aiming at the spatial structure of bacteria populations may be an alternative to changing the cell density. Inversely, spatial organisation will make a bacterial behaviour more effective and protect it from treatment influence.

The presented hypothesis about fitness optimisation on colony level needs further investigation. Our generic basic model does not include any feedback of the regulated behaviour on the autoinducer regulation, nor a fitness analysis. Experiments on the level of energy balance or reproduction success are needed to verify the hypothesis. The general lack of quantitative data and mechanistic insight for the (often nonlinear) relation between multiple environmental factors and autoinducer system activity impedes an detailed understanding of the ecologic purpose of signalling. It can be overcome by differentiated quantitative data from chemostat and retentostat experiments. Furthermore, experiments analysing the spatial pattern within colonies associated with various environmental conditions as well as the interaction of autoinducer systems between colonies and between species in the light of gradient induced heterogeneity are highly desirable.

## 4. Material and Methods

### 4.1. Parameter Choice

We discuss the parameters and the rationales for the choice of a numerical value assigned to it one after the other. For a quick overview, consult Table 2. It is necessary to emphasise that most of the parameters may vary from strain to strain, and even for one single strain under different conditions. Sometimes, only indirect indications about numerical values are available. We selected typical values, but by no means the only possible values. The qualitative results presented in the paper, however, are expected not to depend on the special choice, but to remain valid for a whole range of parameters.

#### 4.1.1. Cell and Colony Geometry

##### Geometry of the Colony

The colony has a radius *R*; this radius is varied in order to investigate the influence of different colony sizes. The cell density within the colony is chosen in such a way that half of the volume is occupied by cells. This is appropriate for *V. fischeri*, the cells of which occupy in light organs up to 60–70% of the volume [29]. We choose *θ* = 0.5, and

$$\rho \left(x\right)=\{\begin{array}{cc} \theta & \text{for}\left|x\right|<R\\ 0 & \text{else} \end{array}$$

We assume the colony not to affect the diffusion rates of all diffusible substances considered here. Biofilm is known to change the diffusion rates, but this effect is rather weak.

##### Cell Volume

The volume of a cell *V_c_* is chosen to be 1 *μm*^3^; the information provided by literature about size of *V. fischeri* varies [30,31], the volume of a cell during the life cycle changes by a factor of three to ten [32].

#### 4.1.2. Parameter for Nutrient

##### Diffusion Rate

The diffusion rate *D_N_* is comparable with that for the autoinducer, assuming that the molecular weight of (some) essential nutrients to be comparable with that of AHL.

*K_m,N_* resp. *K_cat,N_* are chosen roughly in the same range like that for aerobic O_2_ respiration. As we do not address specific nutrients, we only aim at values that are in a reasonable range. We expect the results to vary quantitatively with these parameters, but not qualitatively.

#### 4.1.3. Parameter for Autoinducer

##### Diffusion Rate

The diffusion coefficients for AHL are well known; [33] gives 4.9 × 10^−10^ m^2^/s, the relation between molecular mass and diffusion coefficient given in [34] yields for 3OC6HSL a diffusion coefficient of 9 × 10^−10^ m^2^/s. We choose the latter value.

The determination of the basic production rate, the increased production rate in the induced state, the threshold concentration and the Hill coefficient is more subtle.

##### Threshold

The threshold is around 10 nmol/l [35]. This is in the same magnitude as an estimation for *Pseudomonas putida* [36], where one finds that the threshold is 70 nmol/l.

##### Basic Production Rate

As in literature we mainly find experiments that measure the luminescence and not the AHL concentration directly, we will need to interfere *α* indirectly. Data of batch experiments can be used to link the basic production rate and the threshold. We outline how to do this, check our method with data about *Pseudomonas putida* [36], and finally use data of Gray and Greenberg [37] to come up with a value for the basic production rate for *V. fischeri*.

We consider a cell culture in its exponential growing phase. The cell density (in cells per litre) behaves like

$$p\left(t\right)=p_{0}e^{\text{bt}}$$

Assume that *x*(*t*) is the AHL concentration (in mol/l), *α̃* the basic production rate (in mol/(cell·s)), and *x*(0) = 0. Note that the production rate *α* used in the present paper relates the production to the fraction of the volume occupied by cells, while *α̃* measures the production per cell. Then,

$$\begin{array}{cc} \dot{x}=\tilde{\alpha }p\left(t\right), & x\left(0\right)=0 \end{array}$$

*i.e.*,

$$x\left(t\right)=\frac{p\left(0\right)\tilde{\text{α}}}{b}\left(e^{\text{bt}}-1\right)=\frac{\tilde{\text{α}}}{b}\left(p\left(t\right)-p\left(0\right)\right)$$

The threshold *xˆ* for the AHL concentration is just *x*(*T*), where *T* denotes the time at which the population becomes induced. As *p*(*T*) ≫ *p*(0), we have

$$\hat{x}\approx \tilde{\text{α}}p\left(T\right)/b=\text{α}\tilde{K}$$

with *K̃* = *p*(*T*)/*b*. To check this method, we use data for *Pseudomonasputida* [36], as in that paper AHL has been measured directly, and thus reliable data are available. The results given in this article read

$$\begin{array}{cccc} \tilde{\text{α}}=2.3\times 10^{-19}\text{mol}/\left(\text{cell h}\right), & A_{\tau }=70\;\text{nmol}/\text{l}, & \text{T}=10\text{h}, & p\left(T\right)=1.13\times 10^{11}\text{cells}/\text{l} \end{array}$$

Also the growth rate can be obtained from these data. It roughly reads 0.6/h. Our method yields *K* = 0.19 × 10^−12^ cells h/l, and thus

$$\tilde{\text{α}}\approx A_{\tau }/K=3.7\times 10^{-19}\text{mol}/\left(\text{cell h}\right)$$

This is, our method gives a comparable result, using the population dynamics and time to activation only.

We find from the data of Gray and Greenberg [37],

$$\begin{array}{cc} b=0.32/h, & T=12h,\text{P}\left(12\right)=p\left(12h\right)=180\times 10^{8}\text{cells}/\text{l} \end{array}$$

and thus

$$\text{K}=\frac{180\times 10^{8}\text{cells}/\text{l}}{0.32/h}=5.6\times 10^{10}\frac{\text{cell h}}{l}$$

The parameters *α* and *β* used in the present work do not relate the production rate to cells but to volume. Furthermore, we use s and not h as reference time scale. All in all, we obtain the proportionality factor *K* as

$$K=\tilde{K}V_{c}=5.6\times 10^{10}\frac{\text{cell h}}{l}\left(10^{-18}\frac{{\text{m}}^{3}}{\text{cell}}\right)=0.2\text{s}$$

and

$$\text{α}=A_{\tau }/K=10\;\text{nmol}/\left(0.2\;\text{s l}\right)=50\;\mu \text{mol}/\left(\text{s}\;{\text{m}}^{3}\right)$$

##### Induced Production Rate

We set the maximal production rate *α* + *β* ten times larger than *α*, orientating ourselves rather at the data for *Pseudomonas putida* [36] than [38].

##### Hill Coefficient

The last parameter to define for the AHL system is the Hill coefficient. The Hill coefficient represents the cooperative aspect in the positive feedback loop, mainly determined by the oligomerisation degree of LuxR-AHL complexes. We choose *n* = 2.5 [36].

##### Modulation of AHL Production by Nutrient

We consider two cases. The first scenario assumes no influence by nutrient, *f*(*N*) = 1. Here, the nutrient plays no role at all.

The second scenario takes the effect of nutrient availability on the signalling and the O_2_ consumption pathway into account. In [10], Figure 2, data for the dependence of the activation of the AHL system on nutrient availability are given. We used these data, assuming that 100% Luria broth LB medium is the reference value, thus nutrient availability is set to one in this case. We use the relative amplification factor *f*(*N*) of the form

$$f\left(N\right)=\frac{N_{1}N^{\text{nN},1}}{N_{\text{τ},1}^{2\text{nN}.1}+N^{2\text{nN},1}}+\frac{N_{2}N^{\text{nN},2}}{{\text{N}}_{\text{τ},2}^{,\text{nN}.2}+N^{\text{nN},2}}$$

A fit of 100 · *f*(*N*) (to transform the amplification factor into percents) using the data of [10] yields the parameters

$$\begin{array}{c} N_{1}=0.74\\ N_{\text{τ},1}=0.14\\ n_{N,1}=0.13\\ N_{2}=0.5\\ N_{\text{τ},2}=0.43\\ n_{N,2}=2 \end{array}$$

### 4.2. Numerical Scheme

As the equations for nutrient and AHL possess the same structure, we only consider one prototypic equation.

#### 4.2.1. General Model

For all three reaction-diffusion equations, the structure reads

$$\begin{array}{ll} \upsilon_{t} & =D\Delta \upsilon +\upsilon g\left(\upsilon ,x\right)\\ \upsilon \left(x\right){|}_{\left|x\right|=\infty } & =\upsilon_{0} \end{array}$$

Please note that also a reaction term of Hill type can be written in this form:

$$\begin{array}{cc} g\left(\upsilon ,x\right)=\frac{V_{\max}\upsilon^{n-1}}{{\text{K}}_{M}^{n}+\upsilon^{n}}\Rightarrow & \upsilon g\left(\upsilon ,x\right)=\frac{V_{\max}\upsilon^{n}}{{\text{K}}_{M}^{n}+\upsilon^{n}} \end{array}$$

If we consider the radially symmetric setting, we find

$$\begin{array}{cc} \upsilon_{t} & =D\left(\upsilon \prime \prime +\frac{2}{r}\upsilon \prime \right)+\upsilon g\left(\upsilon ,r\right)\\ \upsilon \left(x\right){|}_{r=\infty } & =\upsilon_{0} \end{array}$$

Now we consider the stationary problem,

$$\begin{array}{l} 0=D\left(\upsilon \prime \prime +\frac{2}{r}\upsilon \prime \right)+\upsilon g\left(\upsilon ,r\right)\\ \upsilon \left(x\right){|}_{r=\infty }=\upsilon_{0} \end{array}$$

It is useful to define the function *w*(*r*) by

$$\upsilon \left(r\right)=\frac{w\left(r\right)}{r}$$

The equation for *w* reads

$$\begin{array}{ccc} 0=D\frac{w\prime \prime }{r}+\frac{w}{r}g\left(w/r,r\right) & \Rightarrow & w\prime \prime =-\text{wg}\left(w/r,r\right)/D \end{array}$$

and *w*′(*r*) *→ ∞_0_* for *r →* ∞. Furthermore, we do not allow for a pole of *υ* at *r* = 0; this yields the boundary condition *w*(0) = 0.

#### 4.2.2. Cut Infinity

Now we restrict the scenario further: we assume *g*(*r*) = 0 for *r* ≥ *R*. Thus, we can solve the equation for *w* explicitly for *r* ≥ *R*,

$$\begin{array}{ccc} w\left(r\right)=a+\upsilon_{0}r & \text{for} & r\geq R \end{array}$$

We derive an equation for *w* on the compact interval [0, *R*]. We already know that *w*(0) = 0. We ensure that *w* ∈ *C*^1^ (at least), *i.e.*,

$$w\prime \left(R\right)=\upsilon_{0}$$

Hence, we find the boundary value problem

$$\begin{array}{ccc} w\prime \prime =-\text{wg}\left(w/r,r\right)/D, & w\left(0\right)=0, & w\prime \left(R\right)=\upsilon_{0} \end{array}$$

This ordinary differential equation can be numerically solved, for given parameters, using a shooting method. Please note that in the original formulation (concentration *υ*) one criterion to select a solution has been the exclusion of a pole for *υ* at the origin. This condition is (especially numerically) rather difficult to use. In this reformulation, the condition that *υ* does not exhibit a pole becomes a boundary condition for *w* (*w*(0) = 0), which leads to a situation where standard methods are feasible.

## Figures and Tables

**Table 1.** State space of the model.

**Variable**	**Meaning**	**unit**
*N*(*t, x*)	Generic nutrient, relative concentration	U
*A*(*t, x*)	AHL concentration	mol/m^3^
*ρ*	Cell concentration (fraction of volume occupied by cells)	1

**Table 2.** Parameters used. The rationales and citations for these choices are given in the text.

**Symbol**	**Meaning**	**Value**	**Units**
*R*	Colony radius	varied, 0 to 70	*μ*m
*θ*	Fraction of volume occupied by cells	0.5	1
*V_c_*	Volume of one cell	1	*μ*m^3^/cell

*D_N_*	Diffusion coefficient for nutrient availability	9 × 10^−10^	m^2^/s
*K_cat_,N*	*K_cat_* for nutrient consumption	10	mol/(m^3^·s)
*K_m_,N*	*K_m_* for nutrient consumption	100 × 10^−6^	mol/m^3^

*D_A_*	Diffusion coefficient for AHL	9 × 10^−10^	m^2^/s
*α*	Basic production of AHL (related to volume)	50	*μ*mol/(m^3^·s)
*β*	Induced production of AHL	450	*μ*mol/(m^3^·s)
*A_τ_*	Threshold	10	nmol/l
*n*	Hill coefficient	2.5	1
*f*(*N*)	Modulation of AHL by nutrient	see text	1
